# UHPLC-ESI-Orbitrap-MS Analysis of Biologically Active Extracts from *Gynura procumbens* (Lour.) Merr. and *Cleome gynandra* L. Leaves

**DOI:** 10.1155/2020/3238561

**Published:** 2020-01-27

**Authors:** Machap Chandradevan, Sanimah Simoh, Ahmed Mediani, Nor Hadiani Ismail, Intan Safinar Ismail, Faridah Abas

**Affiliations:** ^1^Agri-omics & Bioinformatics Programme, Biotechnology & Nanotechnology Research Centre, MARDI, Persiaran MARDI-UPM, 43400 Serdang, Selangor, Malaysia; ^2^Laboratory of Natural Products, Institute of Bioscience, Universiti Putra Malaysia, 43400 Serdang, Selangor, Malaysia; ^3^Atta-ur-Rahman Institute for Natural Product Discovery, Universiti Teknologi MARA, Puncak Alam Campus, 42300 Bandar Puncak Alam, Selangor, Malaysia; ^4^Department of Food Science, Faculty of Food Science and Technology, Universiti Putra Malaysia, 43400 Serdang, Selangor, Malaysia

## Abstract

This study aimed to determine the total phenolic content, DPPH scavenging, *α*-glucosidase, and nitric oxide (NO) inhibition of *Gynura procumbens* and *Cleome gynandra* extracts obtained with five different ethanolic concentrations. The findings showed that the 100% ethanolic extract of *G. procumbens* had the highest phenolic content and the lowest IC_50_ values for DPPH scavenging and NO inhibition activity compared to the properties of the other extracts. For *C. gynandra*, the 20% and 100% ethanolic extracts had comparably high total phenolic contents, and the latter possessed the lowest IC_50_ value in the NO inhibition assay. In addition, the 20% ethanolic extract of *C. gynandra* had the lowest IC_50_ value in the DPPH scavenging assay. However, none of the extracts from either herb had the ability to inhibit *α*-glucosidase enzyme. Pearson correlation analysis indicated a strong relationship between the phenolic content and DPPH scavenging activity in both herb extracts. A moderately strong relationship was also observed between the phenolic content and NO inhibition in *G. procumbens* extracts and not in *C. gynandra* extracts. The UHPLC-ESI-Orbitrap-MS revealed major phenolics from the groups of hydroxycinnamic acids, hydroxybenzoic acids, and flavonoid derivatives from both herbs, which could be the key contributors to their bioactivities. Among the identified metabolites, 24 metabolites were tentatively assigned for the first time from both species of studied herbs. These two herbs could be recommended as prospective natural products with valuable medicinal properties.

## 1. Introduction

Plants and herbs have a history of traditional uses and are important parts of cultural heritage. Their appreciation as food and links to health-promoting benefits are also significant. Since ancient times, herbs have been utilised traditionally to cure many illnesses, which has prompted modern science to fully understand their benefits. *Gynura procumbens* (Asteraceae), locally known as Sambung nyawa, is an annual grown herb that has thick leaves and hardened stems with a slight purple tint during maturation. The young leaves can be eaten raw as salads. Its ethnomedicinal usages are well reported; for example, in Indonesia, *G. procumbens* is used to treat fever, skin rashes, and ringworm infection [1]. In Thailand, it is used to treat inflammation, viral infections, and rheumatism [2]. Scientific investigations on *G. procumbens* include antidiabetic, antihypertensive [3], anticancer [4], and anti-inflammatory [5] studies. In terms of its phytochemical constituents, phenolic compounds, such as kaempferol, quercetin, astragalin [6], kaempferol 3-*O*-rutinoside, rutin, and chlorogenic acid [7], were identified as the key metabolites that contributed to the bioactivities in the reported studies.

*Cleome gynandra* (Cleomaceae), known as maman, is used in ayurvedic treatment, especially for treating lump, prostate enlargement, worm infections, and ear diseases [8]. The leaves of *C. gynandra* are crushed into a concoction and drunk to cure scurvy in Africa [8]. Scientific investigations on the biological activities of *C. gynandra* include antioxidant [9], anti-inflammatory [10], antidiabetic [11], and anticancer [12]. However, phytochemical analysis is still lacking for *C. gynandra*, except in a recent report that listed caffeic acid, coumaric acid, sinapic acid, and ferulic acid as the major phytochemicals in this herb [13]. Ranjitha and colleagues [14] identified *β*-amyrin, *β*-amyrin-3-*O*-*β*-glucopyranoside, sitosterol, and stigmasterol by NMR and GC-MS from this herb. The *C. gynandra* was reported to be rich in minerals, carotenoids, flavonoids, alkaloids, terpenoids, steroids, and tannins.

Inflammation is a coordinated response in the body towards harmful stimuli, such as injuries, pathogenic infection, and allergens. Macrophages are the cells responsible for initiating inflammation by producing inflammatory mediators, such as cytokines, interferons, and nitric oxide (NO) as response to stress [15]. Study has also indicated how the production of excessive inflammatory mediators leads to the onset of diabetes [16]. Among the treatments available for diabetes, the inhibition of *α*-glucosidase is one of them, which is responsible for glucose breakdown in the intestinal wall [17]. Plants' phenolic compounds have been reported to have the ability in inhibiting this enzyme, yet more often, modern drugs can be prescribed to combat the occurrence of inflammation-related diseases and diabetes [18]. However, prolonged use of these drugs stimulates unwanted side effects towards the liver, kidney, and other organs [19]. In return, naturally occurring phytochemicals from herbs have been ventured as an alternative medicine since they possess reduced or no toxicity when consumed at lower doses. *G. procumbens* and *C. gynandra* are two herbs that have the potential to be explored further for their anti-inflammatory and antidiabetic properties based on past studies. An optimum and proper extraction protocol may help researchers study the beneficial health properties of herbs, thus enabling the development of herbal-based products [20]. Extraction protocols that vary based on the type of sample, extraction solvents, temperature, extraction time, and instruments used play important roles in the standardization of herbs [21].

In this study, the efficacy of *G. procumbens* and *C. gynandra* extracted with different ethanol concentrations was tested for *α*-glucosidase and NO inhibition. Nevertheless, detailed metabolite profile and the effect of solvents extractions on distribution of metabolites of both herbs are still lacking. As such, this study proposed to investigate the aforementioned properties of the herbal extracts. The total phenolic content (TPC) and 2,2-diphenyl-1-picrylhydrazyl (DPPH) free radical scavenging activity were also tested as to support the anti-inflammatory and antidiabetic properties of the studied herbs. The relationship between TPC and biological activity was studied using the Pearson correlation model. Active ethanolic extracts from both herbs were then subjected to ultra high-performance liquid chromatography-electron spray ionisation-orbitrap-mass spectrometry (UHPLC-ESI-Orbitrap-MS), and potential metabolites that may contribute to the tested bioactivities were tentatively identified and reported.

## 2. Materials and Methods

### 2.1. Chemicals and Reagents

HPLC-grade ethanol and Folin–Ciocalteu's reagent were purchased from Merck (Darmstadt, Germany). Sodium bicarbonate and dimethyl sulfoxide (DMSO) were supplied by Fisher Scientific (Leicestershire, UK). 2,2-Diphenyl-1-picrylhydrazyl (DPPH), quercetin hydrate, curcumin, gallic acid, 4-nitrophenyl *α*-D-glucopyranoside (PNPG), lipopolysaccharide (LPS), phosphate-buffered saline (PBS), and recombinant murine interferon gamma (IFN-*γ*) were obtained from Sigma-Aldrich (St. Louis, USA). The *α*-glucosidase enzyme was purchased from Megazyme (Bray Business Park, Ireland). The reagents used for cell culture studies, including Dulbecco's Modified Eagle's Medium (DMEM), containing HEPES and L-glutamine, with and without phenol red; an antibiotic mixture of penicillin-streptomycin, fetal bovine serum (FBS), 3-(4,5-dimethylthiazol-2-yl)-2,5-diphenyltetrazolium bromide (MTT), and TripLE™ Express enzyme were supplied by Gibco (Life technologies, USA). The LC/MS graded acetonitrile was purchased from Fisher Scientific (Toronto, Canada). RAW 264.7 murine macrophage cells were obtained from American Type Culture Collection (ATCC, Rockville, MD).

### 2.2. Plant Material

*C. gynandra* was collected from a local farmer in Rompin, Negeri Sembilan. Samples were randomly plucked from plots in an open area that had been preinstalled with automated irrigation. *G. procumbens* was grown and harvested from the net house located in the Malaysia Agricultural, Research and Development Institute (MARDI). Both herbs were collected before noon to maintain their freshness. Upon collection, voucher sample from both herbs were sent to MARDI's herbarium and authenticated by a botanist, Dr. Mohd Norfaizal Ghazalli (voucher specimen numbers for *G. procumbens* and *C. gynandra* are MDI 12841 and MDI 12840, respectively). Only the leaves from both herbs were used in this study. The leaves were washed under running tap water and gently dried using laboratory tissue paper. After that, they were ground with liquid nitrogen using a mortar and pestle and freeze-dried immediately. The dried powder samples were stored at -20 °C prior to analysis.

### 2.3. Extraction of Samples

Six replicates of freeze-dried samples of both *G. procumbens* and *C. gynandra* were extracted with different concentrations of ethanol/water ratios (0, 20, 50, 70, and 100% ethanol). In general, the dried samples (4 g) were mixed in 250 mL conical flasks with 100 mL of each ethanol ratio. The mixture was then homogenised using a homogeniser (Ultra Turrax, IKA, Germany) at 6000 rpm for 1 min followed by shaking in a shaker (ES-20, Biosan, Latvia) at 220 rpm for 15 min at room temperature. The supernatant was filtered with125 mm diameter filter paper (Advantec, Japan). The extraction procedures were repeated twice. The collected supernatant was concentrated using a rotary evaporator (RII, Buchi, Switzerland) and further freeze-dried (Freezone 6, Labconco, USA) to eliminate any moisture. Prior to bioassay analysis, the crude extracts of 0 and 20% ethanol/water were dissolved in deionised water, whereas the others were dissolved in dimethyl sulfoxide (DMSO).

### 2.4. Total Phenolic Content (TPC) Assay

The TPC assay was carried out in accordance with the method described in [22] with minor modifications. Modifications were made in terms of the volume of reagents used in the assay. In general, 20 *μ*L of 350 *μ*g/mL of the extract was mixed with 100 *μ*L of Folin–Ciocalteu's reagent (10-fold dilution) in a 96-well plate. Then, 80 *μ*L of 7.5% sodium carbonate was added, and the mixture was left in the dark for 30 min prior to the absorbance reading at 750 nm (SpectraMax PLUS, USA). Gallic acid was serially diluted ranging from 0.78 to 100 *μ*g/mL and used to make a standard curve in this assay. The results are expressed as mg gallic acid equivalent (GAE) per 100 mg of dried extract.

### 2.5. 2,2-Diphenyl-1-picrylhydrazyl (DPPH) Scavenging Assay

The DPPH scavenging assay was performed based on the method described in [22] with minor modifications to the concentration and volume of DPPH used. In general, 50 *μ*L of extract with serial dilutions ranging from 10.94 to 350 *μ*g/mL was mixed with 100 *μ*L of DPPH (0.15 mM) in a 96-well plate and incubated in the dark for 30 min. The absorbance of the solution was measured at 515 nm. Quercetin was used as the positive control, and all experiments were performed in six replicates. The results are expressed as the concentration (*μ*g/mL) of extract needed to scavenge 50% of DPPH (IC_50_).

### 2.6. *α*-Glucosidase Inhibition Assay

The inhibition of the *α*-glucosidase enzyme by *G. procumbens* and *C. gynandra* extracts was evaluated based on the method described in [23] with minor modifications to the used volume of the enzyme and substrate. Prior to the experiment, *α*-glucosidase and the substrate PNPG were dissolved in 50 mM phosphate buffer at pH 6.5. In brief, 10 *μ*L of plant extract with a serial dilution ranging from 10.94 to 350 *μ*g/mL was mixed with 130 *μ*L of 30 mM phosphate buffer and 10 *μ*L of the enzyme (2 U/mL) in a 96-well plate. After 5 min of incubation, 50 *μ*L of the PNPG substrate was added followed by and reincubation at room temperature for another 15 min. The reaction was ceased by the addition of 50 *μ*L of glycine (pH 10) before the absorbance was measured at 405 nm. Quercetin was used as the positive control in this study. The results are expressed as the concentration (*μ*g/mL) of extract needed to inhibit 50% of *α*-glucosidase (IC_50_).

### 2.7. Nitric Oxide (NO) Inhibition Activity

The NO inhibition assay was performed in accordance with the method described in [24]. The RAW 264.7 cells were grown in culture flasks using phenol-red DMEM under 5% CO_2_ at 37°C. Once confluency reached 80%, cells were detached using 2.5 mL TrypLE™ Express enzyme. Prior to cells seeding in 96-well plates, cells were counted using the standard Trypan blue counting technique, where the cell concentration was set to 1 × 10^4^ cells/mL in all wells. Seeded cells (50 *μ*L/well) were left in an incubator for 24 h before proceeding with induction and treatment. In all, 50 *μ*L (1 *μ*L IFN-*γ* + 1*μ*L LPS + 48 *μ*L DMEM) triggering agent was added into the designated wells, followed by 50 *μ*L of plant extract (serially diluted from 15.63 to 500 *μ*g/mL). Curcumin was used as the positive control in this assay. All analyses were performed in six replicates, and the cells were incubated 17–24 h at 37°C under a 5% CO_2_ atmosphere.

### 2.8. Measurement of Nitrite

A Griess assay was performed to measure the accumulation of nitrite ions (NO_2_^−^), a conversion product from NO in a simple manner. An incubated 96-well plate was removed, and 50 *μ*L of media from the plate was transferred attentively into a new 96-well plate. Then, 50 *μ*L of Griess reagent (1% sulfanilamide, 0.1% N-(1-naphtyl)-ethylene diamine dihydrochloride, and 2.5% phosphoric acid) was added, and the plate was left in the dark for 15 min at room temperature. Sodium nitrite (NaNO_2_) at 200 *μ*M was used as a positive control in this assay. Absorbance at 550 nm was measured after incubation.

### 2.9. Cell Viability Test

Cell viability was assessed by the 3-(4,5-dimethylthiazol-2-yl)-2,5-diphenyltetrazolium bromide (MTT) assay to determine the cytotoxicity of the plant extract. Fresh phenol-red DMEM (100 *μ*L) was added to the wells containing cells, followed by 20 *μ*L of MTT (dissolved in 1× PBS buffer). The plate was then incubated for 4 h at 37°C under a 5% CO_2_ atmosphere. Next, all the media were discarded, and 100 *μ*L of DMSO was added. The plate was left for 15 min in the dark at room temperature before the absorbance was measured at 570 nm. The percent viability of the cells was calculated by comparing the absorbance of the treated cells with the control group (untreated cells).

### 2.10. Metabolite Profiling Using the UHPLC-ESI-Orbitrap-MS

The separation and identification of metabolites from *G. procumbens* and *C. gynandra* were achieved using a UHPLC system (Ultimate 3000™, Thermo Scientific) coupled with mass spectrometry (Q Exactive™ Hybrid Quadrupole-Orbitrap, Thermo Scientific). Separation of the metabolites was performed using a Thermo C18 column (2.1 mm × 100 mm, 1.9 *μ*m) with mobile phases of LCMS-grade acetonitrile with 0.1% formic acid as buffer B and deionised water with 0.1% formic acid as buffer A. The flow rate was set at 274 *μ*L/min, and UV detection was set at 280 nm. The gradient setting of the mobile phases was set for a total run time of 30 min and divided as follows: equilibration of column for 5 min at 95% of solvent A, a steady decrease in solvent A for 20 min until reaching 5%, maintenance of 5 % solvent A for another 5 min, and a steep increase in solvent A to 95% in one minute. The column was re-equilibrated for 4 min at 95% solvent A. All samples were prepared at a concentration of 2.0 mg/mL in 30% methanol and filtered by a nylon syringe filter (0.45 *μ*M, 13 mm diameter). Ten microliters of each sample was injected into the system. Ionization of the metabolites was performed using an ESI probe in negative mode. The capillary temperature was set at 300°C with a scanning range from 50–1500 amu. All analyses were performed and monitored using Xcalibur 2.2 software (Thermo Scientific Inc, Waltham, MA, USA). The identification and characterization of metabolites were performed by relative comparison from previously reported data and from online databases [25, 26]. The mass error in ppm was calculated by comparing the theoretical monoisotopic mass from the online databases to the observed mass.

### 2.11. Statistical Analysis

All bioassay results were reported as the mean of six biological replicates with the standard deviation. One-way analysis of variance (ANOVA) was performed with a significant difference between the collected data set at a confidence interval of 95%. The Pearson correlation test (*r* value) was calculated to evaluate the relationship between the bioactivities. All calculations were performed using GraphPad PRISM version 5.01 for Windows (San Diego, CA, USA).

## 3. Results and Discussion

### 3.1. Total Phenolic Content of *G. procumbens* and *C. gynandra* Extracts

The TPC results of the *G. procumbens* and *C. gynandra* extracts are presented in Table 1. The TPC assay was performed to measure the relative amounts of phenolic compounds from the herbal extracts since most phenolic compounds were found to possess health benefits [27]. *G. procumbens* extracted with 100% ethanol was observed to have the highest phenolic content with 5.91 mg GAE/100 mg dried extract (de). The TPC trend was 100% > 70% > 50% > 20% > 0% ethanolic extract as the ratio of water increased. On the other hand, the TPC of *C. gynandra* extracts showed mixed results, with 0%, 20%, and 100% ethanolic extracts showing significantly high phenolic content with 3.38, 3.48, and 3.71 mg GAE/100 mg de, respectively. The TPC values increased as the ratio of ethanol increased and started to drop in the 50% and 70% ethanolic extracts before rising again at the 100% ethanolic concentration. The difference in the TPC trend shown by the two herbs could be due to the type of metabolites being extracted. Previous study has indicated how polar metabolites in nature have the tendency to be extracted with polar solvents and vice versa [28]. Secondary metabolites, such as tannins, hydroxycinnamic acids, and flavonoids are regarded as polar in nature, whereas sterols, terpenoids, and lipids are semipolar and nonpolar [29]. Water, being a universal solvent, is also more polar than ethanol. Increasing the water ratio in ethanol makes the final concentration of the extraction solvent more polar. In this study, different water/ethanol concentrations were thought to extract metabolites of different polarities, thus giving different clusters of metabolites in each extraction solvent.

### 3.2. DPPH Scavenging Activity of the *G. procumbens* and *C. gynandra* Extracts

The ability of the ethanolic extracts of *G. procumbens* and *C. gynandra* to scavenge DPPH free radicals was tested, and the results are presented in Table 1. Unlike TPC, DPPH scavenging in the *G. procumbens* extracts showed fluctuating results. The 20% ethanolic extract of *C. gynandra* had the lowest IC_50_ value of 40.36 *μ*g/mL, followed by the water extract with IC_50_ value of 62.01 *μ*g/mL. The 50 and 100% ethanolic extracts of *G. procumbens* had the IC_50_ values of 66.28 *μ*g/mL and 63.73 *μ*g/mL, respectively, with no significant difference. The IC_50_ of the other ethanolic extracts were fluctuated showing the trend of 0% > 70% > 20%. In addition, the rest of the ethanolic extracts possessed poor DPPH scavenging activity, as shown in Table 1.

### 3.3. *α*-Glucosidase Inhibition Activity of the *G. procumbens* and *C. gynandra* Extracts

Unlike the rest of the assays, neither extract (*G. procumbens* or *C. gynandra*) showed any inhibition against the *α*-glucosidase enzyme. In this study, extracts from both plants at 10 mg/mL were tested against *α*-glucosidase, and no activity was detected except for the positive control, quercetin. Quercetin showed 92.3% inhibition at 50 *μ*g/mL. This finding contradicts findings from other researchers, for example, Ngwe and colleagues [30] reported that they obtained IC_50_ values of the 95% ethanolic and water extracts of *G. procumbens* to be 1.05 *μ*g/mL and 1.06 *μ*g/mL, respectively. Another study reported that the water extract of *G. procumbens* yielded an IC_50_ of 0.092 mg/mL [31]. Further investigation from the reported studies leads to the observation of certain parameters that were not applied in our studies. *G. procumbens* was extracted thrice with water for 12 h, which was different from our extraction protocol. The incubation temperature during the assay also seemed to play an important role in the enzyme activity [32]. Khatib et al. [29] reported low inhibition of the *α*-glucosidase enzyme using a *Momordica charantia* ethanolic extract and suggested different origin, maturity, postharvest, and processing conditions as possible factors for their results. On the other hand, this is the first ever reported study on the inhibition of *α*-glucosidase of *C. gynandra* ethanolic extracts.

### 3.4. NO Inhibition Activity of the *G. procumbens* and *C. gynandra* Extracts

The *G. procumbens* 70% and 100% ethanolic extracts had significantly high inhibitory effects on NO production, with IC_50_ values of 22.91 *μ*g/mL and 25.20 *μ*g/mL, respectively. The *C. gynandra* extracts also showed remarkable NO production inhibition results. The 70% and 100% ethanolic extracts of *C. gynandra* have the lowest IC_50_ values of 66.01 *μ*g/mL and 60.75 *μ*g/mL, respectively, with significant difference. The inhibitory pattern of both herbs was similar in that the inhibitory activity increased gradually from 0% > 20% > 50% > 70% and 100% ethanolic extracts. The MTT assay further confirmed that none of the *G. procumbens* and *C. gynandra* extracts possessed cytotoxicity towards the cells, which strengthened the possibility for the development of anti-inflammatory drugs.

### 3.5. Correlation between TPC, DPPH Scavenging, and NO Inhibition of *G. procumbens* and *C. gynandra* Extracts

To justify whether phenolic compounds from the studied herbs correlate with DPPH scavenging and NO inhibitory activity, a Pearson correlation test was performed at the 95% confidence interval. No correlation data were obtained for the *α*-glucosidase assay since none of the extracts showed any inhibition towards the enzyme. A strong positive or negative correlation has a scored *r* value between +0.5 and +1.0 or −0.5 and −1.0. If the score is between +0.1 and +0.3 or −0.1 and −0.3, then the association is considered weak [33].

The correlation between TPC and the DPPH scavenging assay showed that the *G. procumbens* extracts scored a strong *r* value of 0.8443, whereas *C. gynandra* scored lower with *r* = 0.5888. This indicates that phenolic compounds could be one of the key contributors to the DPPH scavenging activity in both herbs. DPPH is a stable free radical and is frequently used to test the antioxidant capacity of herbal extracts. Plants' phenolic acids have been proven to be the main source of DPPH scavenging activity [28] in which DPPH free radicals receive electrons from phenolic acids and are reduced to the stable DPPH-H complex [34]. Our finding is also in line with the data presented for *G. procumbens* extracts [35]. The Pearson correlation between TPC and the NO inhibition activity of *G. procumbens* extract showed a moderately strong *r* value at 0.6418. This indicates a possible contribution from phenolic compounds in the inhibition of NO production via RAW 264.7 cell induction. By contrast, the *C. gynandra* extracts showed a weak negative correlation with an *r* value of 0.2432. A weak negative correlation may be due to the TPC assay providing an estimation of the total phenolic compounds presents in an extract. However, nonphenolic compounds, such as ascorbic acid and tocopherol, are also able to reduce Folin–Ciocalteu's reagent [36]. The presence of nonphenolic metabolites in *C. gynandra* extracts could contribute to a higher TPC and may not inhibit NO production.

### 3.6. Tentative Identification and Characterization of Phenolic Compounds from the *G. procumbens* and *C. gynandra* Extracts

**Figure 1:**
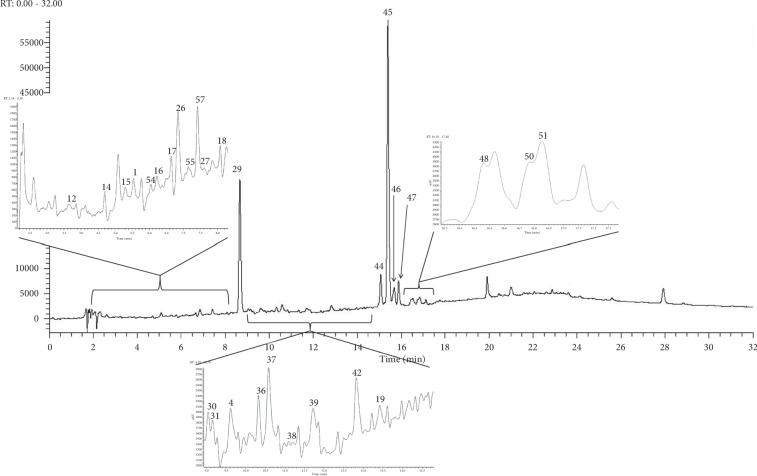
UHPLC chromatogram of 100% ethanolic extract of *G. procumbens* at 280 nm.

**Figure 2:**
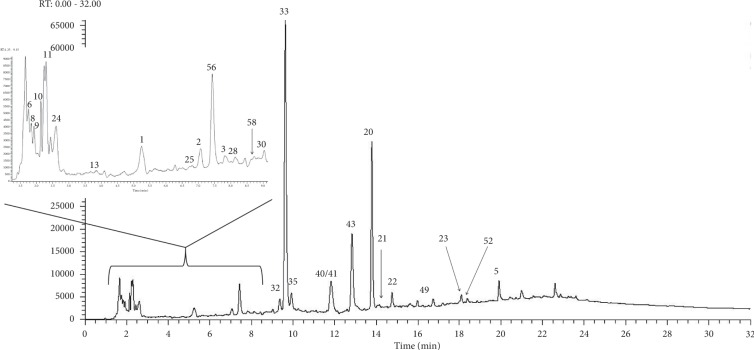
UHPLC chromatogram of 20% ethanolic extract of *C. gynandra* at 280 nm.

**Figure 3:**
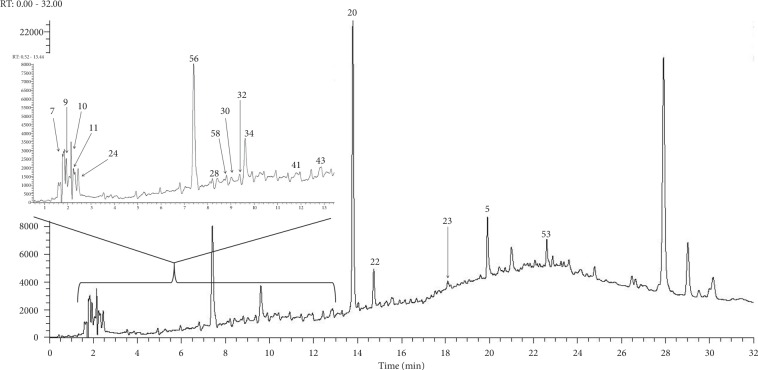
UHPLC chromatogram of 100% ethanolic extract of *C. gynandra* at 280 nm.

The extracts with the best representation of their total phenolic contents, DPPH scavenging activities, *α*-glucosidase inhibition, and nitric oxide (NO) inhibition from both herbs were chosen for analysis by UHPLC-ESI-Orbitrap-MS. Ergo, the 100% ethanolic extract of *G. procumbens* and the 20% and 100% ethanolic extracts of *C. gynandra* were chosen for this purpose.  Table 2 shows the list of tentatively identified phenolic acids grouped according to their class. Among the 58 phenolic acids identified, 27 were found only in the *G. procumbens* extract, and 11 and 3 were found only in the 20% and 100% ethanolic extracts of *C. gynandra*, respectively. The remaining 17 phenolic acids can be found in at least two of the extracts analysed. The theoretical monoisotopic mass and mass error of phenolic acids, which were identified as derivative or dimer, were not determined since information of their actual structure were lacking. This analysis also showed that hydroxycinnamic acids were the major phenolic acids identified in all extracts. Other classes of phenolic acids were also identified, including the hydroxybenzoic acids, flavonoid derivatives, organic acids, and sugar derivatives. Figures 1–3 show the chromatograms of the 100% ethanolic extract of *G. procumbens* and the 20% and 100% ethanolic extracts of *C. gynandra*, respectively.

#### 3.6.1. Hydroxycinnamic Acid Derivatives

A total of 30 phenolic acids within the class of hydroxycinnamic acid and its derivatives were tentatively identified from all extracts. Metabolites **26**, **31,** and **36** were found to have a precursor ion at *m*/*z* 353 [M-H]^−^, further fragmentations yielded product ions at *m*/*z* 191, 179, 173, and 135, which was similar to the ionization pattern of caffeoylquinic acid [38, 41]. The existence of isomers in caffeoylquinic acid has been well documented based on the difference in intensity of the product ions *m*/*z* 179 and 173. Metabolite **31** had an intense signal at *m*/*z* 173 due to the loss of [quinic acid-H-H_2_O]^−^, which was lacking in metabolites **26** and **36** due to the particular stereochemical arrangement of their structures. 4-Caffeoylquinic acid was confirmed to show such fragmentation patterns. Metabolites **26** and **36** were distinguished from each other based on their retention time reported from past study and assigned as 3-caffeoylquinic acid and 5-2caffeoylquinic acid, respectively [42]. Metabolite **29** with precursor ion *m*/*z* 707 [M-H]^−^ was observed as one of the major peaks in the 100% ethanolic extract of *G. procumbens*; it produced product ions similar to caffeoylquinic acid but with a dimeric adduct of itself as described in [44]. As such, metabolite **29** was assigned as a dimer of caffeoylquinic acid.

Metabolites **44-47** and **51** shared a similar precursor ion at *m*/*z* 515 [M-H]^−^ with a base peak at *m*/*z* 353 and common product ions at *m*/*z* 191, 179, 173, 161, and 135. These fragmentation patterns are similar to dicaffeoylquinic acid. Further investigation of MS^3^ revealed that metabolites **44**, **47,** and **51** have *m*/*z* 173 [quinic acid-H-H_2_O]^−^ as the base peak, which indicates the presence of a caffeoyl moiety on the quinic acid at position C-4. The caffeoyl moiety attached at a C position other than C-4 in quinic acid will give a base peak at *m*/*z* 191 [quinic acid]^−^ as shown in metabolites **45** and **46**. Based on the retention time shift in the reverse phase chromatogram, metabolites **44**, **47,** and **51** were assigned as 3,4-dicaffeoylquinic acid, 1,4-caffeoylquinic acid, and 4,5-dicaffeoylquinic acid, respectively [48]. Metabolites **45** and **46** were named 3,5-dicaffeoylquinic acid and 1,5-dicaffeoylquinic acid, respectively, since both metabolites eluted just after 3,4-dicaffeoylquinic acid on a reverse-phase column [49]. Comparing the two metabolites, metabolite **45** is structurally more polar than metabolite **46**, which makes the former elute earlier as well [49].

Metabolites **24, 30, 37, 40, 42,** and **48** have a loss of either coumaric acid (164 amu) or a coumaroyl moiety (146 amu) in their fragmentations, which makes identification easier. Metabolite **30** has a precursor ion *m*/*z* 325 [M-H]^−^ with product ion at *m*/*z* 163 [M-H-glucose]^−^ and 119 [M-H-glucose-CO_2_]^−^ which was confirmed as coumaric acid glucoside [41]. Metabolites **37** and **42** with the deprotonated ion *m*/*z* 337 [M-H]^−^ produced a base peak ion at *m*/*z* 191 [M-H-coumaroyl]^−^, which indicates the presence of quinic acid. Subsequent MS^2^ fragmentations produced ions *m*/*z* 173 [M-H-coumaroyl-H_2_O]^−^ and *m*/*z* 127, which confirmed the identity of metabolites **37** and **42** as coumaroylquinic acid [38, 46]. 5-p-coumaroylquinic acid has a unique fragmentation with *m*/*z* 191 as the base peak compared to other coumaroylquinic acid isomers. Since the report [46] was in accordance with our finding, metabolites **37** and **42** were named trans-5-p-coumaroylquinic acid and cis-5-p-coumaroylqunic acid, respectively. Metabolite **48** adds an additional caffeoyl moiety onto coumaroylquinic acid, which gave the precursor ion *m*/*z* 499 [M-H]^−^. This metabolite was assigned as caffeoyl-coumaroylquinic acid [50]. Metabolites **24** and **40** were assigned as derivatives of coumaric acid since they have unknown adducts on their coumaric acid structure [41].

Metabolite **35** was identified as caffeic acid with precursor ion *m*/*z* 179 [M-H]^−^ and product ions at *m*/*z* 135 [M-H-44]^−^ and 117 [M-H-44-18]^−^ after the loss of a carbon dioxide and a water molecule [42]. The structures of metabolites **25, 28, 49, 52,** and **53** were all associated with caffeic acid. Metabolite **25** loses a glucose moiety from its aglycon and is named caffeic acid glucoside [37, 42]. Metabolite **49** with precursor ion *m*/*z* 387 [M-H]^−^ has a base peak at *m*/*z* 193, which suggests that it is a dimer. Further MS^2^ data produced product ion *m*/*z* 179 [193-CH_3_]^−^ after the elimination of a methyl group. As such, metabolite **49** was labelled as a dimer of caffeic acid methyl ester. The presence of caffeic acid ethyl ester was confirmed in metabolite **52** after the precursor ion *m*/*z* 207 [M-H]^−^ produced product ion *m*/*z* 179 [M-H-29]^−^ due to the elimination of an ethyl moiety. Notably, although both metabolites **49** and **52** were tentatively identified for the first time in *C. gynandra*, the existence of other varieties of caffeic acid esters in herbs also suggests the possible occurrence of metabolites **49** and **52** [51]. Metabolites **28** and **53** were noted as derivatives of caffeic acid since the conjugation of unknown moieties in caffeic acid produced precursor ions *m*/*z* 297 [M-H]^−^ and 599 [M-H]^−^, respectively.

Metabolites **32-34, 41,** and **43** were linked to dimethoxycinnamoyl as they shared the same product ions at *m*/*z* 207 [M-H]^−^, 189 [M-H-H_2_O]^−^, 127 [M-H-H_2_O-2(OCH_3_)]^−^, and 83 [M-H-H_2_O-2(OCH_3_)-CO_2_]^−^ [37]. Metabolites **32** and **34** have a glucose moiety in their structure that gave the precursor ion *m*/*z* 369 [M-H]^−^. Metabolite **33** with precursor ion *m*/*z* 739 [M-H]^−^ indicates a dimeric molecule of dimethoxycinnamoyl glucoside. Metabolites **41** and **43** with precursor ions *m*/*z* 353 [M-H]^−^ and 383 [M-H]^−^, respectively, suggested the addition of a coumaroyl unit (146 amu) and glucuronide (176 amu) in their dimethoxycinnamoyl structure. MS^2^ spectra of metabolite **41** produced product ion *m*/*z* 119 [163-CO_2_]^−^, which further rectified this finding. Other hydroxycinnamic acid derivatives, including sinapic acid glucoside (**27**), feruloylquinic acid (**39**), caffeoyl-feruloylquinic acid (**50**), and derivative of cinnamic acid (**38**) were identified based on previously reported data [38, 40, 43, 46, 47].

#### 3.6.2. Hydroxybenzoic Acid Derivatives

Protocatechuic acid *m*/*z* 153 [M-H]^−^ was associated with metabolites **12**, **15,** and **17** with metabolite **16** identified as protocatechuic acid and had a base peak at *m*/*z* 109 [M-H-CO_2_]^−^ after elimination of a carbon dioxide molecule. Metabolites **12** and **15** are isomers with precursor ion *m*/*z* 315 [M-H]^−^. Elimination of a sugar molecule (162 amu) from the precursor ion produced a base peak similar to that of protocatechuic acid, *m*/*z* 153 [M-H]^−^. As such, metabolites **12** and **15** were labelled as protocatechuic acid glucoside [39]. Metabolite **17** has a deprotonated *m*/*z* 211 [M-H]^−^ with a base peak of 148 [M-H-63]^−^, indicating a loss of an unknown moiety; probably a methoxy (-OCH_3_) and an oxygen molecule. The same precursor ion has a product ion at *m*/*z* 153 [M-H-58]^−^, which could be a loss of a methyl formate moiety (-CO(OCH_3_)), and the MS^2^ data showed *m*/*z* 109 [M-H-58-CO_2_]^−^, similar to protocatechuic acid. Due to the uncertainty of the nature of the adduct, metabolite **17** was assigned as a protocatechuic acid derivative.

Metabolite **13** has a deprotonated ion at *m*/*z* 351 [M-H]^−^ and ion fragments at *m*/*z* 169 after the expulsion of an unknown moiety (182 amu), followed by *m*/*z* 125 [M-H-182-CO_2_]^−^. Metabolite **14**, on the other hand, has a precursor ion *m*/*z* 331 [M-H]^−^ with base peak *m*/*z* 169 [M-H-glucose]^−^ and MS^2^ fragmentation ion 125 [M-H-glucose-CO_2_]^−^. Based on the mass fragmentation pattern, metabolites **13** and **14** were assigned as derivatives of gallic acid and gallic acid glucoside, respectively [40]. Metabolite **18** has a deprotonated ion *m*/*z* 299 [M-H]^−^ and product ion *m*/*z* 137 [M-H-162]^−^ and base peak *m*/*z* 93 [M-H-162-CO_2_]^−^, which confirmed it as hydroxybenzoic acid glucoside [41]. Metabolite **19** has the precursor ion *m*/*z* 280 and base peak at *m*/*z* 119 [M-H-161]^−^. Other product ions of metabolite **19** include *m*/*z* 137 [M-H-143]^−^ and 93 [137-CO_2_]^−^, which represent the possible structure of a hydroxybenzoic acid. As such, metabolite **19** was labelled as a derivative of hydroxybenzoic acid.

#### 3.6.3. Flavonoid Derivatives

Metabolites **20**–**23** were clustered under the flavonoid derivatives. Metabolite **20** has a deprotonated ion at *m*/*z* 609 [M-H]^−^ with a base peak *m*/*z* 301 [M-H-308]^−^ after the elimination of a rutinose moiety. The MS^2^ spectra of *m*/*z* 301 produced *m*/*z* 179 [M-H-308-122]^−^ and 151 [M-H-308-122–28]^−^, which are in line with quercetin ion fragmentations. Thus, metabolite **20** was identified as quercetin rutinoside. Quercetin glucoside was identified as metabolite **21** since it has the precursor ion *m*/*z* 463 [M-H]^−^ and a base peak similar to the quercetin ion after the elimination of a glucose molecule. Metabolite **22** was found to be associated with kaempferol since fragmentation of precursor ion *m*/*z* 593 [M-H]^−^ produced base peak *m*/*z* 285 [M-H-308]^−^ and ion *m*/*z* 151, which is similar to kaempferol rutinoside [38]. Metabolite **23** has a base peak at *m*/*z* 285 [M-H]^−^ after the loss of an unknown mass (330 amu) from the precursor ion *m*/*z* 615 [M-H]^−^. The MS^2^ spectra of base peak *m*/*z* 285 produced ions at *m*/*z* 241 [M-H-44]^−^ and 213 [M-H-44-28]^−^, which are identical to the ions of luteolin [38]. Ergo, metabolite **23** was named as a derivative of luteolin.

#### 3.6.4. Sugar Derivatives

Glucaric acid, *m*/*z* 209 [M-H]^−^, and its ion fragmentations at *m*/*z* 191 [M-H-H_2_O]^−^ and 85 [M-H-2H_2_O-2CO_2_]^−^ were associated with metabolites **1-4** [37]. Metabolite **1** has a caffeoyl moiety conjugated with glucaric acid and is thus identified as caffeoylglucaric acid. In return, metabolites **2-4** have a coumaroyl moiety conjugated in their structure and are labelled coumaroylglucaric acid and an isomer of coumaroylglucaric acid, respectively. Metabolite **5** was identified as a derivative of acetylglucoside since its precursor ion *m*/*z* 465 [M-H]^−^ has a base peak at *m*/*z* 243 after a possible elimination of the acetylglucose moiety (222 amu). Further ion fragmentations produced *m*/*z* 221 [M-H-222-22]^−^ and 177 [M-H-222-22-CO_2_]^−^, which remains unknown.

#### 3.6.5. Organic Acids

Metabolites **6-11** were identified as malic acid, citric acid, and their derivatives, respectively, based on their fragmentation spectra. Malic acid, *m*/*z* 133 [M-H]^−^, had product ions at *m*/*z* 115 [M-H-H_2_O]^−^ and 71 [M-H-H_2_O-CO_2_]^−^, whereas citric acid, *m*/*z* 191 [M-H]^−^ with product ions *m*/*z* 173 [M-H-H_2_O]^−^ and 111 [M-H-2H_2_O-CO_2_]^−^, confirmed their identity [38].

#### 3.6.6. Other Classes of Phenolic Compounds

Other classes of phenolic compounds such as dihydroxybenzene (hydroxytyrosol glucoside **54**), hydroxybenzaldehyde (derivative of syringaldehyde **55**), hydroxycoumarin (esculetin glucoside **56**, esculetin sambubioside **57**), and hydroxyphenylacetic acid (homovanillic acid glucoside **58**) were tentatively identified based on previously reported data [38, 39, 52].

## 4. Conclusions

The DPPH scavenging, *α*-glucosidase inhibition, and NO inhibition activities as well as TPC were tested for *G. procumbens* and *C. gynandra* extracted with different ethanolic concentrations. The results showed a preliminary understanding of the potential of both herbs to serve as antioxidant and anti-inflammatory agents. In total, 58 metabolites were identified in both herbs with 24 metabolites were identified for the first time. Tentatively identified metabolites help to reduce the gap in unknown metabolites from both herbs and are important for future reference. Future studies using both herbs as herbal formulations should investigate the synergistic effects of the metabolites on herbal product development.

## Figures and Tables

**Table 1:** The total phenolic content (TPC), DPPH scavenging activity, nitric oxide (NO) inhibition activity, and cell cytotoxicity (MTT) of the *G. procumbens* and *G. gynandra* extracts.

Extracts % EtOH	*G. procumbens*				*C. gynandra*			
	TPC (mg GAE/100 mgde)	DPPH (IC50) *μ*g/mL	NO inhibition (IC50) *μ*g/mL	MTT (%) at 1000 *μ*g/mL	TPC (mg GAE/100 mgde)	DPPH (IC50) *μ*g/mL	NO inhibition (IC50) *μ*g/mL	MTT (%) at 1000 *μ*g/mL
0	1.44 ± 0.03^a^	124.37 ± 6.28^a^	117.19 ± 10.50^a^	96.31 ± 3.06	**3.38** ± **0.06**^a^	62.01 ± 5.84^a^	210.06 ± 12.25^a^	ND
20	1.58 ± 0.32^a^	317.41 ± 8.32^b^	96.77 ± 7.09^b^	97.91 ± 3.84	**3.46** ± **0.26**^a^	**40.36** ± **5.09**^b^	94.83 ± 10.49^b^	ND
50	3.81 ± 0.04^b^	**66.28** ± **3.95**^c^	82.03 ± 8.95^c^	95.76 ± 8.01	1.51 ± 0.18^b^	276.92 ± 6.42^c^	97.11 ± 9.09^b^	ND
70	3.15 ± 0.19^b^	131.92 ± 8.99^a^	**22.91** ± **6.75**^d^	93.54 ± 8.73	2.74 ± 0.28^c^	304.64 ± 9.02^d^	**66.01** ± **7.75**^c^	91.53 ± 3.25
100	**5.91** ± **0.62**^c^	**63.73** ± **2.90**^c^	**25.20** ± **6.19**^d^	91.71 ± 7.72	**3.71** ± **0.26**^a^	236.95 ± 7.96^c^	**60.75** ± **6.32**^c^	89.11 ± 4.77
Quercetin	—	1.73 ± 0.12	—	—	—	1.73 ± 0.12	—	—
Curcumin	—	—	3.59 ± 0.28	—	—	—	3.59 ± 0.28	—

Values are the mean ± standard deviation of six biological replicates. Bold numbers to show extracts with the best activity for related assays. GAE: gallic acid equivalent; de: dried extract; EtOH: ethanol; ND: not detected. Each different superscript letter within the same assay indicates a statistically significant different (*P* < 0.05).

**Table 2:** Tentative identification of phenolic compounds from the extracts of *G. procumbens* and *C. gynandra*.

No	*T*_R_ (min)	**Measured mass[M-H]** ^−^ *m*/*z*	**Theoretical mass[M-H]** ^−^ *m*/*z*	Mass error (ppm)	MS/MS*m*/*z* (% intensity)	Tentatively identified metabolites	**100% GP**	**20% CG**	**100% CG**	Reference(s)
*Sugar derivatives*										
**1**	5.55	371.0621	371.0693	−19.4	209 (20), 191 (20), 179 (5), 173 (5), 85 (100)	**Caffeoylglucaric acid**	+	+	−	[37]
**2**	7.07	355.0671	355.0744	−20.6	209 (4), 191 (3), 163 (4), 119 (5), 85 (100)	**Coumaroylglucaric acid**	−	+	−	[37]
**3**	7.82	355.0673	355.0744	−20.0	209 (40), 191 (10), 163 (3), 133 (8), 119 (4), 85 (100)	Isomer of coumaroylglucaric acid	−	+	−	[37]
**4**	9.62	355.0673	355.0744	−20.0	337 (1), 209 (20), 191 (20), 173 (3), 163 (3), 85 (100)	Isomer of coumaroylglucaric acid	+	−	−	[37]
**5**	19.91	465.2257	n.d	n.d	261 (6), 243 (100), 221 (70), 177 (3), 149 (2)	**Derivative of acetylglucose**	−	+	+	[37]

*Organic acids*										
**6**	1.76	275.0216	n.d	n.d	159 (20), 227 (1), 133 (1), 115 (100), 71 (25)	Derivative of malic acid	−	+	−	[38]
**7**	1.78	217.9913	n.d	n.d	133 (15), 125 (10), 115 (99), 89 (10), 71 (100)	Derivative of malic acid	−	−	+	[38]
**8**	1.83	289.0177	n.d	n.d	133 (60), 115 (100), 71 (35)	Derivative of malic acid	−	+	−	[38]
**9**	1.93	133.0211	133.0215	−3.0	115 (40), 71 (100)	**Malic acid**	−	+	+	[38]
**10**	2.15	405.0248	n.d	n.d	191 (40), 173 (10), 129 (5), 111 (100), 87 (20)	Derivative of citric acid	−	+	+	[38]
**11**	2.30	191.0189	191.0270	−42.4	173 (10), 111 (50), 87 (100), 85 (30), 67 (30), 57 (55)	**Citric acid**	−	+	+	[38]

*Hydroxybenzoic acid derivatives*										
**12**	3.61	315.0724	315.0794	−22.2	153 (80), 109 (100)	**Protocatechuic acid glucoside**	+	−	−	[39]
**13**	3.55	351.0599	n.d	n.d	198 (10), 180 (4), 169 (20), 125 (60), 111 (100) 79 (60)	Derivative of gallic acid	−	+	−	[40]
**14**	4.53	331.0672	331.0743	−21.4	313 (6), 169 (100), 150 (40), 125 (98)	**Gallic acid glucoside**	+	−	−	[40]
**15**	5.30	315.0724	315.0794	−22.2	152 (80), 108 (100)	Isomer of protocatechuic acid glucoside	+	−	−	[39]
**16**	6.23	153.0269	153.0266	1.96	123 (10), 109 (80), 108 (100), 97 (10), 91 (15), 81 (10), 65 (13)	Protocatechuic acid	+	−	−	[39]
**17**	6.64	211.0607	n.d	n.d	153 (60), 148 (100), 138 (12), 136 (15), 123 (10), 120 (20), 109 (70), 108 (40), 95 (20)	Protocatechuic acid derivative	+	−	−	[39]
**18**	8.27	299.0772	299.0845	−24.4	137 (60), 93 (100)	Hydroxybenzoic acid glucoside	+	−	−	[41]
**19**	13.42	280.0649	n.d	n.d	217 (6), 145 (40), 137 (20), 119 (100), 117 (15), 93 (15)	Derivative of hydroxybenzoic acid	+	−	−	[41]

*Flavonoid derivatives*										
**20**	13.80	609.1464	609.1534	−11.5	343 (4), 301 (100), 179 (5), 151 (4)	Quercetin rutinoside	−	+	+	[38]
**21**	14.28	463.0884	463.0877	1.5	301 (50), 300 (100), 271 (5), 255 (3), 179 (4), 175 (2), 151 (4)	**Quercetin glucoside**	−	+	−	[38]
**22**	14.74	593.1516	593.1585	−11.6	327 (3), 285 (100), 151 (2)	**Kaempferol rutinoside**	−	+	+	[38]
**23**	18.26	615.2463	n.d	n.d	571 (10), 553 (6), 386 (4), 285 (100), 241 (4), 213 (1)	**Derivative of luteolin**	−	+	+	[38]

*Hydroxycinnamic acid derivatives*										
**24**	2.44	248.0539	n.d	n.d	180 (20), 163 (60), 153 (10), 119 (100), 107 (15), 93 (20), 72 (50)	Derivative of coumaric acid	−	+	+	[41]
**25**	6.80	341.0881	341.0951	−20.5	179 (70), 135 (100)	Caffeic acid glucoside	−	+	−	[42]
**26**	6.85	353.0879	353.0951	−20.4	191 (100), 179 (50), 161 (4), 135 (80), 111 (4), 93 (2), 85 (4)	3-Caffeoylquinic acid	+	−	−	[38, 41, 42]
**27**	7.56	385.0718	385.0713	1.29	223 (40), 205 (60), 147 (10), 129 (30), 111 (60), 101 (20), 85 (100)	Sinapic acid glucoside	+	−	−	[43]
**28**	8.13	297.0616	n.d	n.d	179 (1), 161 (10), 135 (100), 117 (10), 89 (20)	Derivative of caffeic acid	−	+	+	[42]
**29**	8.66	707.1831	n.d	n.d	353 (20), 191 (100), 179 (1), 161 (1)	Dimer of caffeoylquinic acid	+	−	−	[44]
**30**	9.04	325.0930	325.1002	−22.1	163 (20), 119 (100)	Coumaric acid glucoside	+	+	+	[41]
**31**	9.16	353.0879	353.0951	−20.4	191 (80), 179 (50), 173 (70), 161 (10), 135 (100), 111 (20), 93 (30)	4-Caffeoylquinic acid	+	−	−	[41]
**32**	9.37	369.0369	369.0365	1.08	207 (10), 189 (25), 127 (100), 83 (50)	**Dimethoxycinnamoyl glucoside**	−	+	+	[45]
**33**	9.63	739.1000	n.d	n.d	369 (12), 207 (15), 189 (100), 127 (20)	**Dimer of dimethoxycinnamoyl glucoside**	−	+	−	[45]
**34**	9.63	369.0364	369.0365	−0.27	207 (2), 189 (10), 127 (100), 99 (20), 83 (80)	**Dimethoxycinnamoyl glucoside**	−	−	+	[45]
**35**	9.92	179.0341	179.0423	−45.8	135 (100), 117 (8), 107 (10), 89 (15)	Caffeic acid	−	+	−	[37, 42]
**36**	10.33	353.0879	353.0951	−20.4	191 (100), 179 (1), 173 (2), 161 (3), 127 (2), 111 (1), 85 (4)	5-Caffeoylquinic acid	+	−	−	[38, 41, 42]
**37**	10.58	337.0931	337.1002	−21.1	191 (100), 173 (5), 163 (20), 127 (3), 119 (20), 111 (10), 93 (50)	*trans*-5-*p*-Coumaroylquinic acid	+	−	−	[46]
**38**	11.09	321.1015	n.d	n.d	321 (20), 241 (2), 175 (2), 147 (20), 119 (2), 103 (4), 97 (100)	Derivative of cinnamic acid	+	−	−	[47]
**39**	11.71	367.1034	367.1107	−19.9	191 (100), 173 (30), 155 (4), 134 (20), 111 (20), 93 (50)	Feruloylquinic acid	+	−	−	[38, 46]
**40**	11.83	375.0332	n.d	n.d	185 (50), 163 (30), 134 (10), 127 (25), 119 (100), 103 (10), 101 (40), 83 (20)	Derivative of coumaric acid	−	+	−	[41]
**41**	11.83	353.0409	353.0404	1.41	207 (1), 189 (10), 127 (100), 119 (5), 99 (20), 83 (90)	**Dimethoxycinnamoyl coumaric acid**	−	+	+	[45]
**42**	12.82	337.0930	337.1002	−21.4	191 (100), 173 (2), 163 (2), 127 (2), 119 (2), 93 (4)	*cis*-5-*p*-Coumaroylquinic acid	+	−	−	[38, 46]
**43**	12.84	383.0620	383.0736	−30.3	207 (1), 189 (10), 127 (100), 99 (20), 83 (80)	**Dimethoxycinnamoyl glucoronide**	−	+	+	[45]
**44**	15.07	515.1193	515.1267	−14.4	353 (30), 335 (20), 191 (40), 179 (90), 173 (100), 161 (20), 135 (20)	3,4-Dicaffeoylquinic acid	+	−	−	[48]
**45**	15.38	515.1195	515.1267	−14.4	353 (20), 191 (100), 179 (75), 173 (4), 161 (4), 135 (10)	3,5-Dicaffeoylquinic acid	+	−	−	[49]
**46**	15.66	515.1196	515.1267	−14.2	353 (20), 191 (100), 179 (80), 173 (15), 161 (5), 135 (10)	1,5-Dicaffeoylquinic acid	+	−	−	[49]
**47**	15.86	515.1196	515.1267	−14.2	353 (25), 191 (30), 179 (80), 173 (100), 161 (2), 155 (6), 135 (10)	1,4-Dicaffeoylquinic acid	+	−	−	[48]
**48**	16.43	499.1019	499.1002	3.4	337 (3), 191 (8), 173 (6), 163 (100), 119 (6)	**Caffeoyl-coumaroylquinic acid**	+	−	−	[50]
**49**	16.75	387.1086	n.d	n.d	193 (100), 179 (3), 161 (8), 133 (4)	**Dimer of caffeic acid methyl ester**	−	+	−	[42, 51]
**50**	16.77	529.1353	529.1424	−13.4	367 (8), 193 (100), 179 (4), 173 (8)	**Caffeoyl-feruloylquinic acid**	+	−	−	[40]
**51**	16.85	515.1196	515.1267	−14.2	353 (30), 191 (40), 179 (80), 173 (100), 155 (10), 135 (10)	4,5-Dicaffeoylquinic acid	+	−	−	[48]
**52**	18.39	207.0657	207.0736	−38.2	179 (2), 161 (30), 135 (90), 133 (100), 117 (2), 106 (3), 89 (2)	**Caffeic acid ethyl ester**	−	+	−	[42, 51]
**53**	22.61	559.3124	n.d	n.d	339 (3), 277 (100), 253 (20), 235 (2), 179 (1), 161 (2)	Derivative of caffeic acid	−	−	+	[42]

*Other classes of phenolic acid*										
**54**	6.04	315.1087	315.1158	−22.5	153 (60), 123 (100)	**Hydroxytyrosol glucoside**	+	−	−	[39]
**55**	7.03	403.0915	n.d	n.d	343 (30), 241 (20), 181 (4), 166 (3), 151 (6), 139 (4), 111 (80), 97 (100)	**Derivative of syringaldehyde**	+	−	−	[39]
**56**	7.40	339.0721	339.0794	−21.5	225 (2), 203 (2), 177 (100), 133 (10)	**Esculetin glucoside**	−	+	+	[52]
**57**	7.42	471.1146	471.1217	−15.1	177 (100), 133 (10)	**Esculetin sambubioside**	+	−	−	[52]
**58**	8.72	343.1035	343.0906	−37.6	181 (100), 137 (70), 121 (8), 109 (10)	**Homovanillic acid glucoside**	−	+	+	[38]

GP: *Gynura procumbens*, CG: *Cleome gynandra*, n.d: not determined. (−) and (+) indicate absence or presence of the metabolite in the extract. Bold metabolites indicate metabolites identified tentatively for the first time in the respective herbs.

## Data Availability

The data used to support the findings of this study are available from the corresponding author upon request.
